# Association of Malnutrition, Left Ventricular Ejection Fraction Category, and Mortality in Patients Undergoing Coronary Angiography: A Cohort With 45,826 Patients

**DOI:** 10.3389/fnut.2021.740746

**Published:** 2021-09-16

**Authors:** Ziling Mai, Zhidong Huang, Wenguang Lai, Huanqiang Li, Bo Wang, Sumei Huang, Yingming Shi, Sijia Yu, Qizheng Hu, Jin Liu, Lingyu Zhang, Yong Liu, Jiyan Chen, Yan Liang, Shilong Zhong, Shiqun Chen

**Affiliations:** ^1^School of Biology and Biological Engineering, South China University of Technology, Guangzhou, China; ^2^Department of Cardiology, Guangdong Provincial Key Laboratory of Coronary Heart Disease Prevention, Guangdong Cardiovascular Institute, Guangdong Provincial People's Hospital, Guangdong Academy of Medical Sciences, Guangzhou, China; ^3^Center of Scientific Research, Maoming People's Hospital, Maoming, China; ^4^Department of Cardiology, Maoming People's Hospital, Maoming, China; ^5^Department of Public Health, Guangdong Medical University, Dongguan, China; ^6^Department of Pharmacy, Guangdong Provincial People's Hospital, Guangdong Academy of Medical Sciences, Guangzhou, China

**Keywords:** malnutrition, left ventricular ejection fraction category, all-cause mortality, interaction, population attributable risk

## Abstract

**Background:** The regulatory effect of the left ventricular ejection fraction (LVEF) categories on the association of malnutrition and all-cause mortality in patients undergoing coronary angiography (CAG) have not been adequately addressed.

**Methods:** Forty-five thousand eight hundred and twenty-six patients consecutively enrolled in the Cardiorenal ImprovemeNt (CIN) study (ClinicalTrials.gov NCT04407936) from January 2008 to July 2018 who underwent coronary angiography (CAG). The Controlling Nutritional Status (CONUT) score was applied to 45,826 CAG patients. The hazard ratios of mortality across combined LVEF and/or malnutrition categories were estimated by Cox regression models. Variables adjusted for in the Cox regression models included: age, gender, hypertension (HT), DM, PCI, coronary artery disease (CAD), low-density lipoprotein cholesterol (LDL-C), high-density lipoprotein cholesterol (HDL-C), triglyceride (TRIG), chronic kidney disease (CKD), statins, atrial fibrillation (AF), anemia, and stroke. Population attributable risk (PAR) was estimated for eight groups stratified by nutritional status and LVEF categories.

**Results:** In our study, 42,181(92%) of patients were LVEF ≥ 40%, of whom, 41.55 and 9.34% were in mild and moderate or severe malnutrition status, respectively, while 46.53 and 22.28% in mild and moderate or severe malnutritional status among patients with LVEF < 40%. During a median follow-up time of 4.5 years (percentile 2.8–7.1), 5,350 (11.7%) patients died. After fully adjustment, there is no difference of mortality on malnutrition in LVEF < 40% group (mild, moderate and severe vs. normal, HR (95%CI): [1.00 (0.83–0.98)], [1.20 (0.95–1.51)], [1.41 (0.87–2.29)], respectively, *p* for trend =0.068), but malnutrition was related to markedly increased risk of mortality in LVEF ≥ 40% group (mild, moderate, and severe vs. normal, HR (95%CI): [1.21 (1.12–1.31)], [1.56 (1.40–1.74)], and [2.20(1.67–2.90)], respectively, *p* for trend < 0.001, and p for interaction < 0.001). Patients with LVEF ≥ 40% had a higher malnutrition-associated risk of mortality and a higher PAR than those with LVEF < 40%.

**Conclusions:** Malnutrition is common in CAG patients and it has a greater effect on all-cause mortality and a higher PAR in patients with LVEF ≥ 40% than LVEF < 40%.

## Introduction

Malnutrition is a common complication of several chronic illnesses, and it could accelerate the progression of the disease as part of a vicious cycle relevant to cytokine activation (1–4). Previous studies have shown that malnutrition is an important poor prognostic factor for chronic heart failure (HF) (5), advanced heart failure (AHF) (3), acute decompensated heart failure (ADHF) (6), and preserved ejection fraction (HFpEF) (7).

Current evidence has shown that poor cardiac function was related to increased production of appetite suppression, catabolic cytokines, and muscle catabolism (3, 7–9). Patients with poor cardiac function are more likely to lose appetite and have worse digestion and absorption, which can aggravate malnutrition to affect prognosis. As a result, it may lead to the stereotype that patients with good cardiac function will be considered at low risk of morbidity and mortality from malnutrition. But limited data exist on the prognostic impact of malnutrition focused on patients with good cardiac function. The relationship between nutritional status, good cardiac function and all-cause mortality has not been adequately addressed. Whether the association between malnutrition and mortality differs in patients with or without poor cardiac function is unknown. Understanding the potential interplay of the prognostic impact of malnutrition focused on patients with different cardiac functions may allow more personalized management of patients with or without poor cardiac function.

Left ventricular ejection fraction (LVEF) is reliable measurement for cardiac function evaluation (10, 11). Accordingly, our study aims to explore the relationship between malnutrition and mortality in patients with LVEF ≥ 40% and with LVEF < 40% assessed by the CONUT score in a cohort of patients undergoing coronary angiography (CAG).

## Method

### Data Sources and Study Population

The Cardiorenal ImprovemeNt (CIN) study is a retrospective observational study that enrolled 88,939 consecutive patients undergoing coronary angiography (CAG) or percutaneous coronary intervention (PCI) in Guangdong Provincial People's Hospital, Guangdong, China, hospitalized between January 2008 to December 2018 (ClinicalTrials.gov NCT04407936). PCI was performed according to standard clinical practice guidelines. We excluded patients with missing data on follow-up and missing data of LVEF and did not meet the CONUT score. Eventually, 45,826 patients were included (Supplementary Figure 1). All traceable personal identifiers were erased before analysis to cover patient data confidentiality. The study was conducted according to the declaration of Helsinki and was approved by the Guangdong Provincial People's Hospital ethics committee.

### Baseline Data Collection

Data were obtained from the electronic clinical management records system of the Guangdong Provincial People's Hospital. We had access to all primary and secondary medical records to view the baseline information of patients, which included demographic characteristics, comorbidities, laboratory tests, and medications at discharge. Blood samples except lipid profiles were collected at admission or before CAG and PCI. The lipid measurement was taken after an overnight fasting blood sample.

### Endpoint and Clinical Definition

The primary endpoint was all-cause death which was monitored and recorded by experienced nurses and research assistants through outpatient interviews and telephones. The Controlling Nutritional Status (CONUT) score, as an assessment for the nutritional status of hospitalized patients, was originally proposed in 2005 by Ignacio de Ulibarri et al. (12). The tool incorporates serum albumin (g/L), cholesterol (mmol/L), and total lymphocyte count (10^9^/L) to assess the state of malnutrition. Left ventricular ejection fraction (%) was evaluated in light of the current international recommendations. The estimated glomerular filtration rate (eGFR) was calculated by using the Modification of Diet in Renal Disease (MDRD) formula. Chronic kidney disease (CKD) was defined as eGFR <60 mL/min/1.73 m^2^ (13). Acute myocardial infarction (AMI), hypertension, and diabetes mellitus (DM) were defined using ICD-10 codes. Anemia was defined as a hematocrit ≤ 39% for males or ≤ 36% for females.

### Statistical Analysis

Baseline characteristics are presented as means ± SDs for continuous variables, and proportions for categorical variables, and medians and interquartile ranges (IQRs) for non-normally distributed data. Continuous variables were tested for normal distribution by use of Kolmogorov-Smirnov test. Differences in two categories of LVEF (< 40% and ≥ 40%) in baseline characteristics were compared through the use of Student *t*-test for continuous variables, chi-square tests for categorical variables, and Kruskal-Wallis test for non-normally distributed data. Time-to-event data were shown in graphs using Kaplan-Meier curves. The survival of each group was compared by log-rank test. Cox regression models were used to calculate the hazard ratio (HR) and 95% confidence intervals (CIs) for mortality across combined LVEF and/or malnutrition categories, respectively. Models were adjusted for age, gender, hypertension (HT), DM, PCI, coronary artery disease (CAD), low-density lipoprotein cholesterol (LDL-C), high-density lipoprotein cholesterol (HDL-C), triglyceride (TRIG), CKD, statins, atrial fibrillation (AF), anemia, and stroke. The Wald chi-square test was used to estimate the *P*-value of the interaction between LVEF categories and malnutrition. R (ver. 4.0.3) was used in all statistical analyses.

## Result

### Patient Characteristics

**Figure 1:**
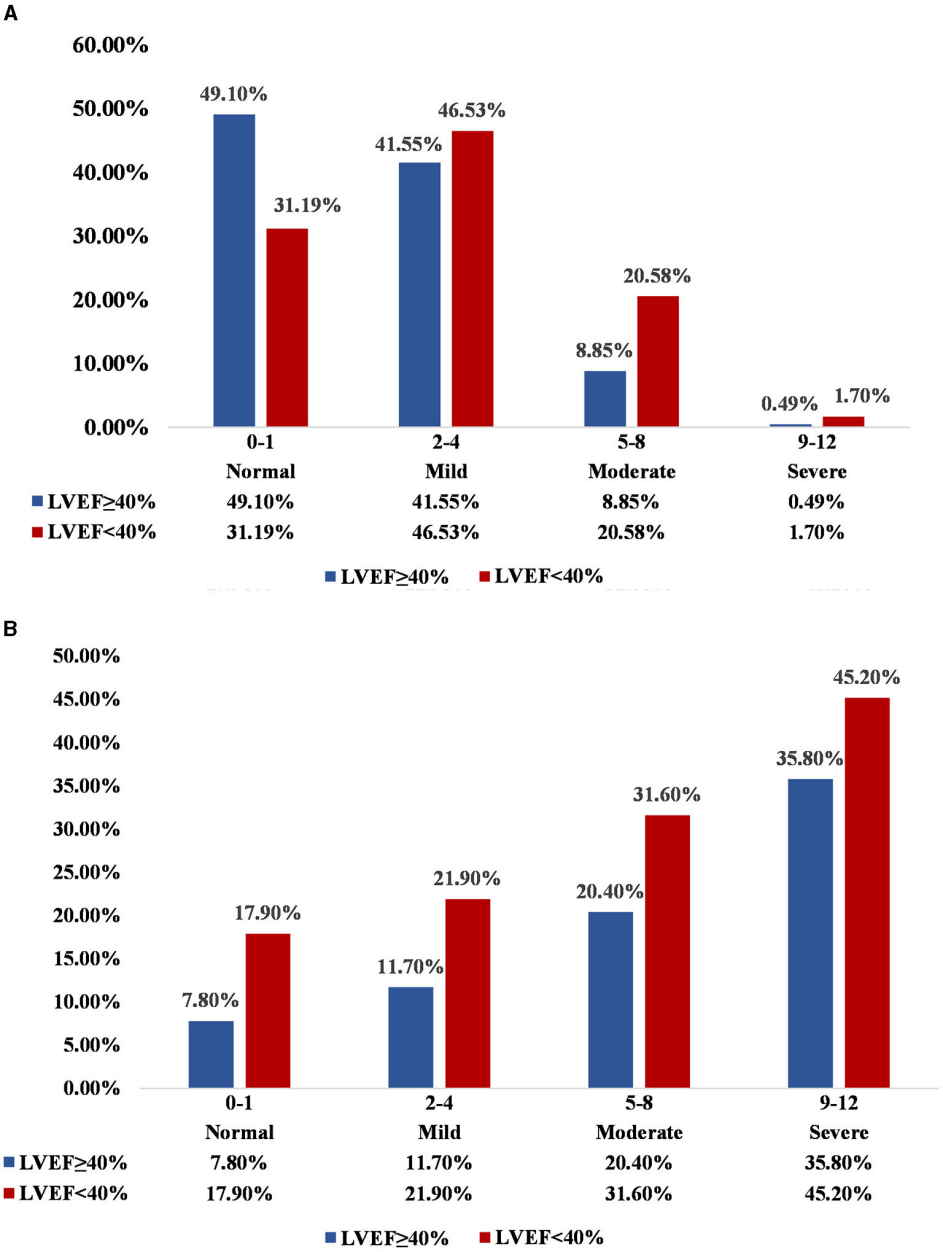
**(A)** Distribution of nutritional state in LVEF ≥ 40% and LVEF < 40%; **(B)** Incidence of death across malnutrition and LVEF categories. Normal, Mild, Moderate, and Severe correspond to the state of malnutrition, respectively, based on the CONUT score. Normal: CONUT 0–1; Mild: CONUT 2–4; Moderate: CONUT 5–8; Severe: CONUT 9–12.

Among the 45,826 patients undergoing coronary angiography enrolled, the mean age was 61.9 ± 10.3 years, 30,942 (67.5%) patients were male, 5,909 (12.9%) had AMI, 13,591 (29.7%) had anemia and a total of 21,621 (47.2%) who underwent PCI treatment. Most patients with LVEF ≥ 40% were female; used antihypertensive medications; had higher serum total cholesterol (CHOL), TRIG, HDL-C, albumin (ALB), lymph cell count (LYMPH1), Na and eGFR, and lower lipoprotein(a), white blood cell (WBC), URIC, aspartate aminotransferase (AST), pre-operative creatinine (pre-CREA) and N-terminal pro–B-type natriuretic peptide (NT-proBNP). There were no apparent differences in AF, hemoglobin (HGB) and LDL-C in the two groups. Of the patients with LVEF < 40%, mild malnutrition accounted for 46.5% and moderate to severe malnutrition for 22.3% by CONUT, while there were 41.6% in mild malnutritional status and 9.3% were in moderate to severe malnutritional status among patients with LVEF ≥ 40%. In general, the malnutrition status of the LVEF ≥ 40% group was better (Figure 1A). More information on the baseline characteristics of patients enrolled was presented in Table 1.

**Table 1:** Baseline characteristics of study patients across LVEF categories.

**Characteristics**	**Overall cohort**	**LVEF ≥ 40%**	**LVEF < 40%**	***P*-value**
	***n* = 45,826**	***n* = 42,181**	***n* = 3,645**	
**Demographic characteristics**				
Age, year	61.9 ± 10.3	61.8 ± 10.3	62.7 ± 10.9	<0.001
Male, *n* (%)	30,942 (67.5)	28,041 (66.5)	2,901 (79.6)	<0.001
**Medical history**				
AMI, *n* (%)	5,909 (12.9)	5,244 (12.4)	665 (18.3)	<0.001
CHF, *n* (%)	4,870 (10.6)	3,601 (8.5)	1,269 (34.8)	<0.001
Anemia, *n* (%)	13,591 (29.7)	12,282 (29.1)	1,309 (35.9)	<0.001
Hypertension, *n* (%)	22,729 (49.6)	21,120 (50.1)	1,609 (44.2)	<0.001
DM, *n* (%)	10,153 (22.2)	9,041 (21.4)	1,112 (30.5)	<0.001
PCI, *n* (%)	21,621 (47.2)	19,629 (46.5)	1,992 (54.7)	<0.001
CKD, *n* (%)	7,485 (16.3)	6,259 (14.8)	1,226 (33.6)	<0.001
COPD, *n* (%)	347 (0.8)	303 (0.7)	44 (1.2)	<0.001
AF, *n* (%)	4,394 (9.6)	4,053 (9.6)	341 (9.4)	<0.001
**Laboratory tests**				
Lipoprotein(a), mg/dL	151.34 [82.00, 318.16]	149.00 [80.92, 312.42]	184.57 [98.94, 389.00]	<0.001
WBC, 10^9^/L	7.68 ± 2.55	7.63 ± 2.51	8.16 ± 2.90	<0.001
HGB, g/L	133.14 ± 16.88	133.18 ± 16.63	132.74 ± 19.51	0.132
CHOL, mmol/L	4.59 ± 1.18	4.60 ± 1.18	4.48 ± 1.20	<0.001
TRIG, mmol/L	1.61 ± 1.17	1.62 ± 1.19	1.44 ± 0.88	<0.001
APOB, g/L	0.86 ± 0.24	0.86 ± 0.24	0.88 ± 0.24	0.002
LDL-C, mmol/L	2.84 ± 0.94	2.84 ± 0.94	2.85 ± 0.98	0.564
HDL-C, mmol/L	1.03 ± 0.28	1.04 ± 0.28	0.97 ± 0.27	<0.001
HbA1c, %	6.37 ± 1.30	6.34 ± 1.27	6.69 ± 1.48	<0.001
URIC, μmol/L	400.65 ± 115.76	394.96 ± 110.62	466.74 ± 148.82	<0.001
ALB, g/L	36.77 ± 4.17	36.93 ± 4.10	34.85 ± 4.59	<0.001
LVEF, %	60.22 ± 12.06	62.69 ± 8.84	31.60 ± 5.80	<0.001
LYMPH, 10^9^/L	1.96 ± 0.74	1.98 ± 0.74	1.77 ± 0.70	<0.001
Na, mmol/L	139.15 ± 2.94	139.23 ± 2.88	138.31 ± 3.40	<0.001
AST, IU/L	24.00 [20.00, 33.00]	24.00 [20.00, 32.00]	27.00 [21.00, 42.00]	<0.001
ALT, IU/L	22.00 [16.00, 33.00]	22.00 [16.00, 33.00]	25.00 [17.00, 41.00]	<0.001
pre-CREA, μmoI/L	84.80 [71.08, 101.00]	83.70 [70.60, 99.00]	99.00 [83.00, 123.56]	<0.001
NT-proBNP, pg/ml	250.50 [63.19, 1083.00]	195.50 [56.84, 821.40]	2577.00 [1162.00, 5582.00]	<0.001
eGFR, mL/min/1.73m^2^	78.84 ± 24.12	79.90 ± 23.81	67.51 ± 24.50	<0.001
**Medications**				
ACEI or ARB, *n* (%)	17,589 (39.5)	15,729 (38.3)	1,860 (52.9)	<0.001
Beta-blockers, *n* (%)	30,545 (68.5)	27,749 (67.6)	2,796 (79.5)	<0.001
Statins, *n* (%)	33,730 (75.7)	31,022 (75.6)	2,708 (77.0)	<0.001
Follow-up date, day	1893.03 ± 1090.91	1911.1 ± 1082.81	1683.43 ± 1160.41	<0.001
CONUT group, *n* (%)				
Normal	21,849 (47.68)	20,712 (49.10)	1,137 (31.19)	<0.001
Mild	19,223 (41.95)	17,527 (41.55)	1,696 (46.53)	
Moderate	4,485 (9.79)	3,735 (8.85)	750 (20.58)	
Severe	269 (0.59)	207 (0.49)	62 (1.70)	

*Values are, n (%), mean ± SDs, or median [IQRs]. AMI, acute myocardial infarction; CHF, congestive heart failure; DM, diabetes mellitus; PCI, percutaneous coronary intervention; CKD, chronic kidney disease; AF, atrial fibrillation; COPD, chronic obstructive pulmonary disease; WBC, white blood cell; HGB, hemoglobin; CHOL, serum total cholesterol; TRIG, triglycerides; APOB, apolipoprotein B; LDL-C, low-density lipoprotein cholesterol; HDL-C, high-density lipoprotein cholesterol; HbA1c, glycosylated hemoglobin; ALB, albumin; LVEF, left ventricular ejection fraction; LYMPH, lymph cell count; Na, sodium,; AST, aspartate aminotransferase; ALT, alanine aminotransferase; pre-CREA, pre-operative creatinine; NT-proBNP, N-terminal pro–B-type natriuretic peptide; eGFR, estimated glomerular filtration rate; ACEI or ARB, angiotensin-converting enzyme inhibitor or angiotensin receptor blocke; CONUT, Controlling Nutritional Status. CONUT scores: CONUT 0-1, normal nutritional status; CONUT 2–4, mildly malnourished; CONUT 5–8, moderately malnourished; CONUT 9–12, severely malnourished*.

### Malnutrition, LVEF Categories, and Mortality

**Figure 2:**
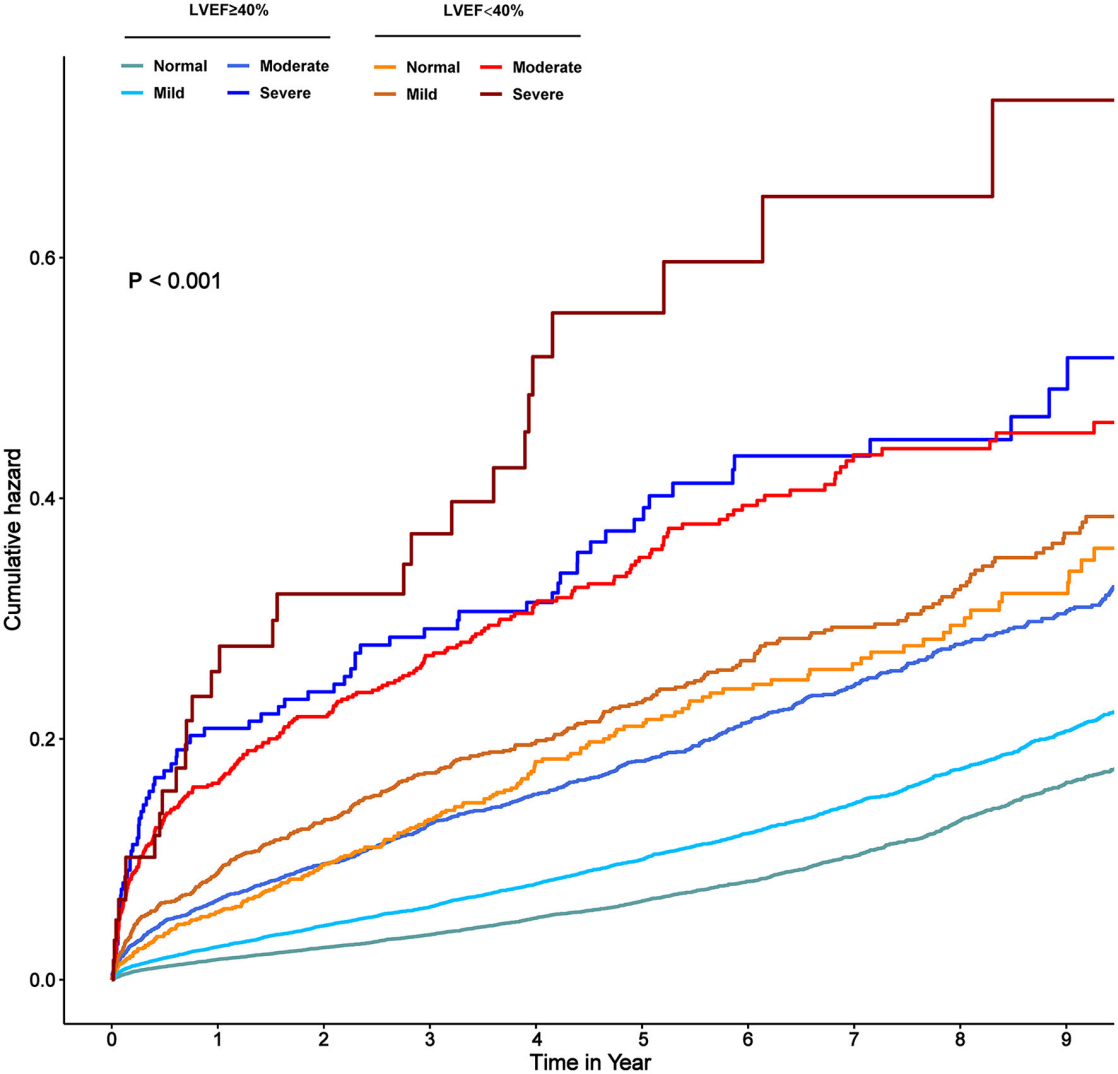
Kaplan–Meier curve in terms of all-cause mortality in normal, mildly, and moderate to severe malnourished patients with and without LVEF < 40%. Normal, Mild, Moderate, and Severe correspond to the state of malnutrition, respectively, based on the CONUT score. Normal: CONUT 0–1; Mild: CONUT 2–4; Moderate: CONUT 5–8; Severe: CONUT 9–12.

During a median follow-up time of 4.5 years (percentile 2.8–7.1), 5,350 (11.7%) patients died. The mortality of normal, mild, moderate and severe malnutrition was 17.9, 21.9, 31.6, and 45.2%, respectively, in patients with LVEF < 40%, while the mortality of normal, mild, moderate and severe malnutrition was 7.8, 11.7, 20.4, and 35.8%, respectively, in patients with LVEF ≥ 40% (Figure 1B). The Kaplan-Meier curve for the relationship of all-cause mortality across nutritional states categorized by LVEF was shown in Figure 2. Increasing the severity of malnutrition demonstrated consistently higher mortality in patients with LVEF ≥ 40% and with LVEF < 40%.

**Figure 3:**
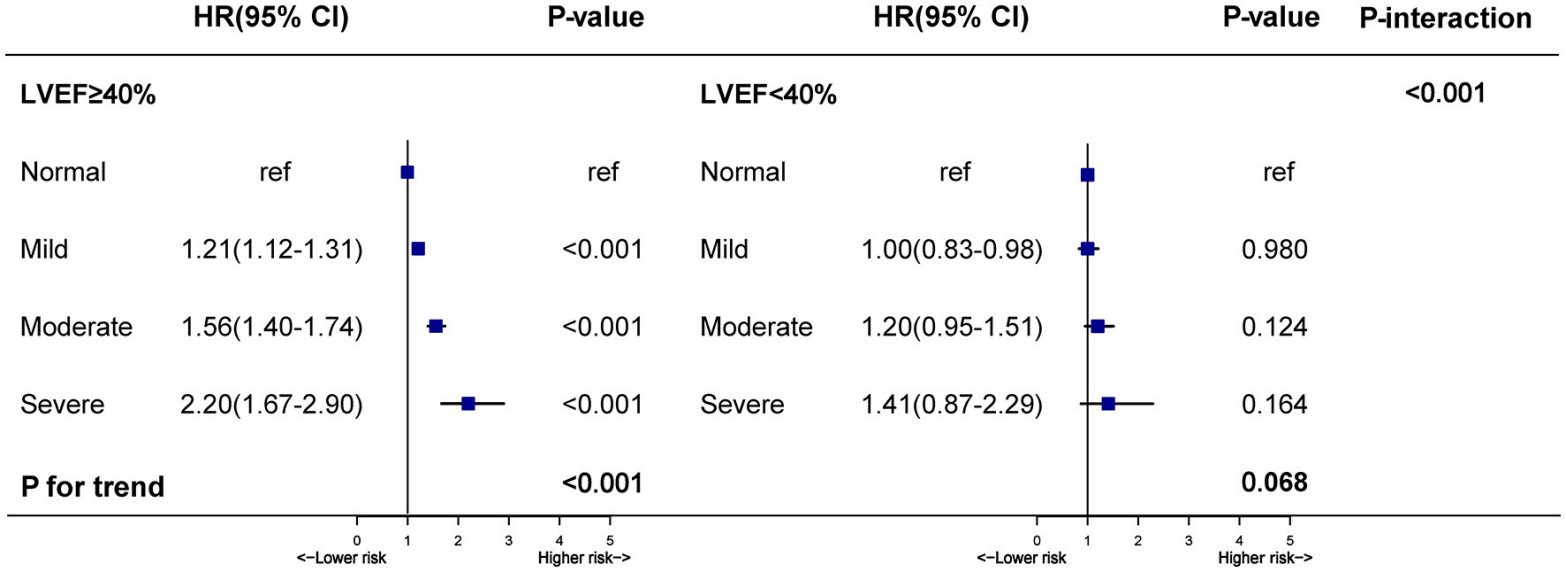
Hazard ratios for all-cause mortality stratified by the nutritional state in patients with LVEF ≥ 40% and LVEF < 40%. Model adjusted for age, gender, percutaneous coronary intervention; hypertension; diabetes mellitus; anemia; stroke; coronary artery disease; chronic kidney diseases; atrial fibrillation; low-density lipoprotein cholesterol; high-density lipoprotein cholesterol; triglycerides and statins. *p-value for interaction test: 2-way interaction of malnutrition (normal vs. mild, moderate, and severe) were severely malnourished and LVEF categories (LVEF ≥ 40% vs. LVEF < 40%).

**Figure 4:**
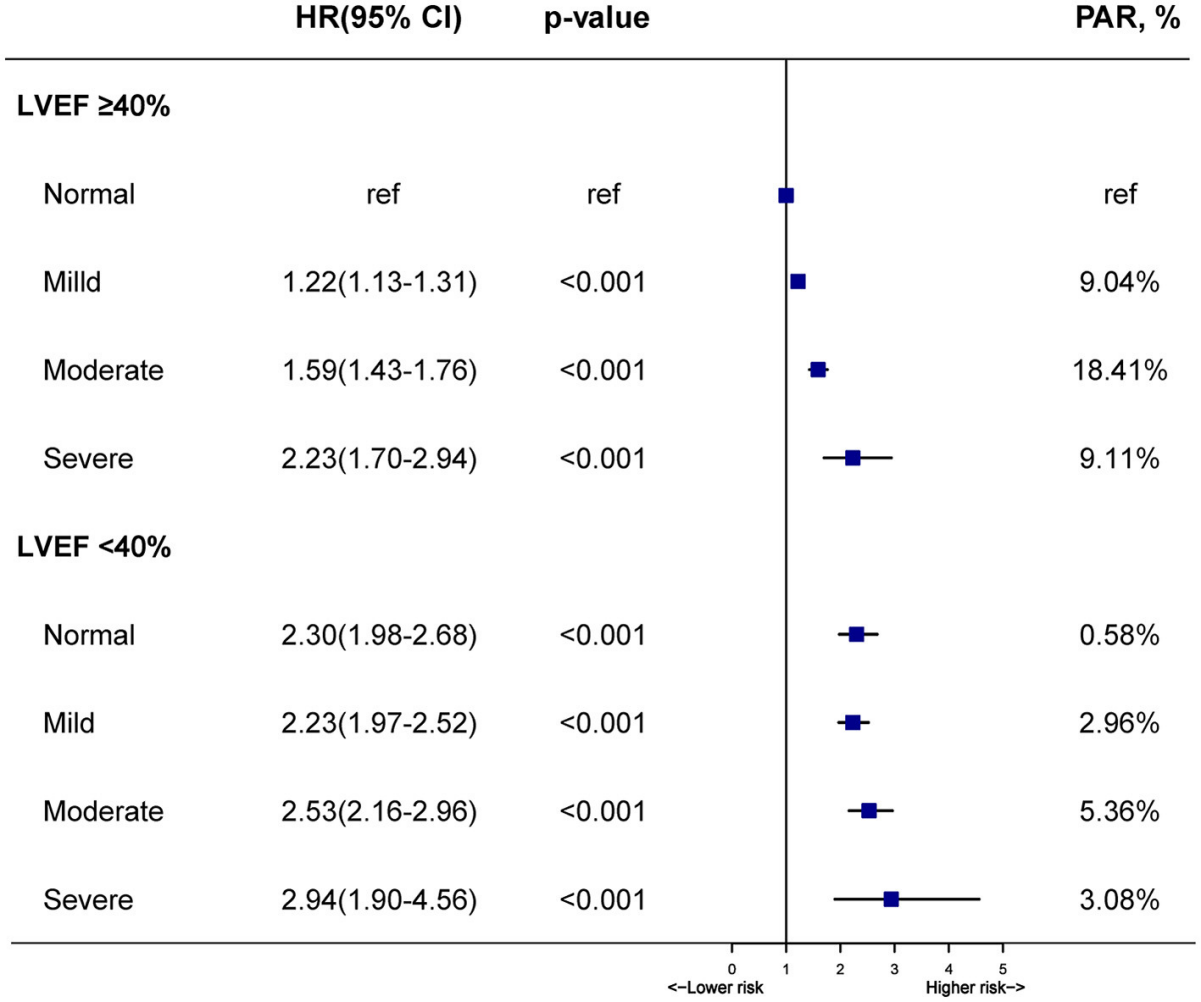
Hazard ratios and population-attributable risk for all-cause mortality across malnutrition and LVEF categories. Model adjusted for age, gender, percutaneous coronary intervention, hypertension, diabetes mellitusc, anemia, stroke, coronary artery disease, chronic kidney diseases, atrial fibrillation, low-density lipoprotein cholesterol, high-density lipoprotein cholesterol, triglycerides and statins.

Controlling for confounding variables, worsening malnutrition status was also associated with a marked upward trend of mortality in patients with LVEF ≥ 40% (mild, moderate, and severe vs. normal, HR: [1.21 (1.12–1.31)], [1.56 (1.40–1.74)], [2.20 (1.67–2.90)], respectively, *p* for trend < 0.001). Although there seemed to be a mildly upward trend in LVEF < 40% group, it was not significant (mild, moderate, and severe vs. normal, HR: [1.00 (0.83–0.98)], [1.20 (0.95–1.51)], [1.41 (0.87–2.29)], respectively, *p* for trend > 0.05). In our study, the highest risk of mortality was present in malnourished patients with LVEF < 40%. However, it was unexpected that patients with LVEF ≥ 40% had a higher malnutrition-associated risk of mortality than those with LVEF < 40% (*p* for interaction < 0.001) (Figure 3). More details of the individual contribution to mortality of all the other variables tested in the multivariate models were shown in Supplementary Table 1. PAR for all-cause mortality in CAG patients was greater for different degrees of malnutrition in LVEF ≥ 40% (normal [ref], mild [9.0%], moderate [18.4%] and severe malnutrition [9.1%]) than those in LVEF < 40% (normal [0.6%], mild [3.0%], moderate [5.4%] and severe malnutrition [3.1%]) (Figure 4).

## Discussion

To our knowledge, this is so far the first largest real-world study to analyze the correlation of malnutrition and all-cause mortality in different LVEF categories. The findings from the present study were that malnutrition was common in CAG patients, and the relationship was unparallel between nutritional status and all-cause mortality in LVEF ≥ 40% and LVEF < 40%. Degrees of malnutrition can stratify the risk of mortality in patients with LVEF ≥ 40%, while malnutrition stratification did not appear to predict mortality risk in patients with LVEF < 40%. This inspired that nutritional intervention may be more effective in patients with relatively good cardiac function.

The first issue to highlight is the prevalence of malnutrition. We chose to study the CONUT score, which is considered as an effective tool for identifying malnutrition in patients (12). The main advantage of this score lies in the fact that it uses only albumin, cholesterol and lymphocyte levels to calculate, which can be computed from parameters readily available on routine testing, eliminating the need for anthropometric measurements. Based on the CONUT score, we discovered a large proportion of our patients suffered from malnutrition. Sixty-nine percent of patients with LVEF < 40% were malnutrition (47% in mild malnutrition status, 22% in moderate to severe malnutrition status) and 51% were in malnutritional status (42% in mild malnutrition, 9% in moderate to severe malnutrition) among patients with LVEF ≥ 40%. Previous studies had reported that malnutrition was an important comorbidity in patients with poor cardiac function. Agra Bermejo et al. (1) indicated that 67% of patients with heart failure suffered from malnutrition using the CONUT score among 145 Spanish with an average age of 69.6 years. Iwakami et al. also showed that 78% of patients with an average age of 75 years from Japan were in bad nutritional status based on the CONUT score among a cohort of 635 AHF patients (14). However, few studies pay attention to the prevalence of malnutrition in patients with relatively good cardiac function currently. In our research, a high prevalence of malnutrition was discovered in patients with LVEF ≥ 40%. Nearly half of them were in a malnourished status, suggesting that malnutrition may be one of the most important comorbidities for patients with relatively good cardiac function. Therefore, our research supports the necessity of malnutrition screening in all patients hospitalized for CAG with LVEF ≥ 40% and with LVEF < 40%. Attention should be paid not only to the nutritional status of patients with LVEF < 40% but also to that in patients with LVEF ≥ 40%.

The second issue that needs to be assessed is the relationship between malnutrition and mortality. Surprisingly, degrees of malnutrition were still related to all-cause death in CAG patients with LVEF ≥ 40% after controlling for confounders in our research. According to studies conducted by Sze et al. (15), worsening malnutrition was associated with worse outcomes in British patients with HF using three scoring systems (Geriatric Nutritional Risk Index—GNRI, the CONUT score and Prognostic Nutritional Index—PNI). Additionally, Minamisawa et al. (7) also proved that malnutrition evaluated by the GNRI was an important factor affecting all-cause death which should not be neglected in patients with HFpEF. However, previous studies were only focused on the relation between malnutrition and mortality in patients with poor cardiac function. Indeed, we found that malnourished patients have a higher mortality rate than those with normal nutrition among patients with LVEF ≥ 40%. Although the highest risk of mortality was present in malnourished patients with LVEF < 40%, it is important to note that patients with LVEF ≥ 40% have a higher malnutrition-associated risk of mortality. What's more, in our study, we found that variables with significant hazard ratios in the Cox regression analysis between different LVEF categories were also different, among of which, gender, CAD, LDL-C, Statins, AF, and Stroke variables had significant hazard ratios in the Cox regression analysis in LVEF ≥ 40% group, but not in the LVEF < 40% group. This suggests that we may need to pay more attention to these variables with significant hazard ratios in patients with LVEF ≥ 40%. Clinical malnutrition screening and intervention should be attached great importance to CAG patients with LVEF ≥ 40% and with LVEF < 40%.

The mechanistically plausible explanations for the association among malnutrition, cardiac function and mortality in patients with CAG are as follows. For patients with poor cardiac function, LV dysfunction caused the release of natriuretic peptides (16), which stimulated lipolysis of adipose tissue (17), and indirectly stimulated the secretion of adiponectin to promote glucose and fatty acid utilization (18), resulting in weight loss and increased burden of death. Metabolites and cytokines released under malnutrition may adversely affect cardiac performance (19) which may also contribute to mortality. Another likely explanation for the high mortality of malnutrition is that nutritional status may correlate with inflammation (20, 21). The increased risk of mortality is because chronic inflammatory diseases were connected with muscle catabolism, catabolic cytokines, albumin consumption and appetite suppression (21). In addition, frailty may be a potentially important link mediating an association between malnutrition and poor health outcomes (22). The likely explanation for the interaction between LVEF categories and malnutrition-associated mortality in our study is that patients with LVEF < 40% at baseline were more likely to be weaker and their conditions at baseline were poorer, thereby associating with a relatively high level of mortality. The impact of malnutrition may be hidden by stronger competing risk factors (LVEF < 40%) for mortality. Owing to the effect, the contribution of malnutrition to prognosis was relatively reduced, and the influence of malnutrition-related mortality was relatively flat. As for those with relatively good cardiac function, their all kinds of body functions were more active, more likely to respond to changes in malnutrition, and more vulnerable to changes in malnutrition, which may cause a rapid increase in the risk of mortality in malnutrition.

Based on our findings, clinicians are strongly recommended to conduct early identification, preventive treatment, nursing management and pharmacologic treatment for the impact of malnutrition on prognosis in CAG patients with different categories of LVEF (23). Screening CAG patients with different categories of LVEF for malnutrition in hospital may identify patients at high risk of adverse outcomes to help them tailor individual treatment on time. At present, the main recommendations of intervention measures in the guidelines for malnourished patients include changing diet, enhancing exercise, and nutritional supplements (24). For malnourished patients with LVEF ≥ 40%, malnutrition may be more easily improved and they are more likely to benefit after improvement. A number of multidisciplinary strategies encourage these patients to accept oral nutritional supplements, food/fluid fortification or enrichment, dietary counseling, and educational interventions to improve their malnutrition state (25). For malnourished patients with LVEF < 40%, heart treatment is mainly taken and nutrient supplement is the secondary auxiliary means. Entresto is approved by United States Food and Drug Administration (US FDA) for heart failure treatment. It is verified as an effective therapy in treating heart failure with reduced LVEF (26). Similarly, CRT and SGLT2i are two effective therapies for HFrEF which can improve the quality of life as well as reduce the rate of heart failure hospitalizations and mortality (27, 28). Moreover, physicians should keep up with the current scientific evidence to combine with their clinical experience to offer the most advantageous, personalized, and optimal protective treatment.

## Limitation

Nevertheless, there are some limitations to this study. First, because it is a single-center, observational study, our findings didn't reflect direct causation. We must always be aware of the potential for residual and uncontrolled confounding which may explain the correlation to some extent. Due to the observational study design, it is necessary to conduct a prospective clinical trial. Unfortunately, information on socioeconomic characteristics, height, weight and/or body composition that might help us understand malnutrition in multiple dimensions was not available. Second, it's essential to further compare the value of the CONUT score tool on the prognosis of patients with other comprehensive malnutrition scoring tools for the reason that malnutrition is a complex problem, especially in elders, which is caused by a variety of factors. Furthermore, our data did not collect information on causes of death, so we cannot directly determine which causes of mortality were directly related to malnutrition. Finally, we only included Chinese individuals that might be restricted regarding generalization across ethnicities; however, we are not aware of whether any data in this study are applicable for other people of most ethnicities. More studies from different countries with other health management and social systems are indispensable to confirm these findings by other researchers.

## Conclusion

Malnutrition is common in CAG patients with LVEF ≥ 40% and LVEF < 40%. Unexpectedly, our findings indicate that malnutrition has a greater effect on prognosis and a higher PAR in patients with LVEF ≥ 40% than LVEF < 40%. Greater attention needs to be given to malnutrition in patients with LVEF ≥ 40%. The findings can be translated into further researches to optimize the outcomes at risk stratification through malnutrition and the LVEF category.

## Data Availability Statement

The datasets generated and analyzed during the current study are not publicly available due to the institution policy but are available from the corresponding author on reasonable request.

## Ethics Statement

The studies involving human participants were reviewed and approved by Guangdong Provincial People's Hospital ethics committee and the study was performed according to the declaration of Helsinki. The ethics committee waived the requirement of written informed consent for participation because our review was a retrospective study of data reuse.

## Author Contributions

ZM, ZH, and WL: research idea and study design. HL and BW: data acquisition. QH: data analysis/interpretation. SC: statistical analysis. SC, SZ, and YLia: supervision and mentorship. JL, SC, YLiu, and JC: writing guidance. All authors contributed important intellectual content during manuscript drafting or revision and accepts accountability for the overall work by ensuring that questions on the accuracy or integrity of any portion of the work are appropriately investigated and resolved, read and approved the final version.

## Funding

This research was funded and supported by the National Key Research and Development Program of China, Grant (2016YFC1301202), Multi-center study on key techniques for prevention, diagnosis and treatment of high risk coronary artery disease (DFJH2020026), Study on the function and mechanism of the potential target for early warning of cardiorenal syndrome after acute myocardial infarction based on transformism (DFJH201919), Natural Science Foundation of Guangdong Province General Project (2020A1515010940), and Guangdong Provincial Science and Technology Plan Project (2017B030314041). The funders had no role in the study design, data collection, and analysis, decision to publish, or preparation of the manuscript, the work was not funded by any industry sponsors.

## Conflict of Interest

The authors declare that the research was conducted in the absence of any commercial or financial relationships that could be construed as a potential conflict of interest.

## Publisher's Note

All claims expressed in this article are solely those of the authors and do not necessarily represent those of their affiliated organizations, or those of the publisher, the editors and the reviewers. Any product that may be evaluated in this article, or claim that may be made by its manufacturer, is not guaranteed or endorsed by the publisher.

## Supplementary Material

Supplementary Figure 1 Flow of patients through the study. CONUT, Controlling Nutritional Status score; LVEF, Left ventricular ejection fraction.

Supplementary Table 1 Hazard ratios for all-cause mortality stratified by the nutritional state in patients with LVEF ≥ 40% and LVEF < 40%.

## References

[1] Agra Bermejo RM González Ferreiro R Varela Román A Gómez Otero I Kreidieh O Conde Sabarís P. Nutritional status is related to heart failure severity and hospital readmissions in acute heart failure. Int J Cardiol. (2017) 230:108–14. 10.1016/j.ijcard.2016.12.067 28038805

[2] Gullett NP Hebbar G Ziegler TR. Update on clinical trials of growth factors and anabolic steroids in cachexia and wasting. Am J Clin Nutr. (2010) 91:1143–7. 10.3945/ajcn.2010.28608E 20164318 PMC2844687

[3] Rahman A Jafry S Jeejeebhoy K Nagpal AD Pisani B Agarwala R. Malnutrition and cachexia in heart failure. J Parenter Enteral Nutr. (2016) 40:475–86. 10.1177/0148607114566854 25634161

[4] Vest AR Chan M Deswal A Givertz MM Lekavich C Lennie T. Nutrition, obesity, and cachexia in patients with heart failure: a consensus statement from the Heart Failure Society of America Scientific Statements Committee. J Card Fail. (2019) 25:380–400. 10.1016/j.cardfail.2019.03.007 30877038

[5] Al-Najjar Y Clark AL. Predicting outcome in patients with left ventricular systolic chronic heart failure using a nutritional risk index. Am J Cardiol. (2012) 109:1315–20. 10.1016/j.amjcard.2011.12.026 22335857

[6] Maeda D Kanzaki Y Sakane K Ito T Sohmiya K Hoshiga M. Prognostic impact of a novel index of nutrition and inflammation for patients with acute decompensated heart failure. Heart Vessels. (2020) 35:1201–8. 10.1007/s00380-020-01590-4 32219523

[7] Minamisawa M Seidelmann SB Claggett B Hegde SM Shah AM Desai AS. Impact of malnutrition using geriatric nutritional risk index in heart failure with preserved ejection fraction. JACC Heart Fail. (2019) 7:664–75. 10.1016/j.jchf.2019.04.020 31302049 PMC13537618

[8] Jones RV. Fat-malabsorption in congestive cardiac failure. Brit Med J. (1961) 1:1276–8. 10.1136/bmj.1.5235.1276 13790589 PMC1954131

[9] Field S Kelly SM Macklem PT. The oxygen cost of breathing in patients with cardiorespiratory disease. Am Rev Respir Dis. (1982) 126:9–13. 7091914 10.1164/arrd.1982.126.1.9

[10] Driessen MM Kort E Cramer MJ Doevendans PA Angevaare MJ Leiner T. Assessment of LV ejection fraction using real-time 3D echocardiography in daily practice: direct comparison of the volumetric and speckle tracking methodologies to CMR. Neth Heart J. (2014) 22:383–90. 10.1007/s12471-014-0577-1 25143268 PMC4160459

[11] Hanachi M Dicembre M Rives-Lange C Ropers J Bemer P Zazzo JF. Micronutrients deficiencies in 374 severely malnourished anorexia nervosa inpatients. Nutrients. (2019) 11:792. 10.3390/nu11040792 30959831 PMC6520973

[12] Ignacio de Ulíbarri J González-Madroño A de Villar NG González P González B Mancha A. CONUT: a tool for controlling nutritional status. First validation in a hospital population. Nutr Hosp. (2005) 20:38–45. 15762418

[13] Levey AS Bosch JP Lewis JB Greene T Rogers N Roth D. A more accurate method to estimate glomerular filtration rate from serum creatinine: a new prediction equation. Modification of Diet in Renal Disease Study Group. Ann Intern Med. (1999) 130:461–70. 10.7326/0003-4819-130-6-199903160-00002 10075613

[14] Iwakami N Nagai T Furukawa TA Sugano Y Honda S Okada A. Prognostic value of malnutrition assessed by Controlling Nutritional Status score for long-term mortality in patients with acute heart failure. Int J Cardiol. (2017) 230:529–36. 10.1016/j.ijcard.2016.12.064 28041709

[15] Sze S Zhang J Pellicori P Morgan D Hoye A Clark AL. Prognostic value of simple frailty and malnutrition screening tools in patients with acute heart failure due to left ventricular systolic dysfunction. Clin Res Cardiol. (2017) 106:533–41. 10.1007/s00392-017-1082-5 28204965

[16] Pellicori P Goode KM Nicholls R Ahmed D Clark AL Cleland JG. Regional circulatory distribution of novel cardiac bio-markers and their relationships with haemodynamic measurements. Int J Cardiol. (2016) 210:149–55. 10.1016/j.ijcard.2016.02.081 26946041

[17] Polak J Kotrc M Wedellova Z Jabor A Malek I Kautzner J. Lipolytic effects of B-type natriuretic peptide 1-32 in adipose tissue of heart failure patients compared with healthy controls. J Am Coll Cardiol. (2011) 58:1119–25. 10.1016/j.jacc.2011.05.042 21884948

[18] Yamauchi T Kamon J Minokoshi Y Ito Y Waki H Uchida S. Adiponectin stimulates glucose utilization and fatty-acid oxidation by activating AMP-activated protein kinase. Nat Med. (2002) 8:1288–95. 10.1038/nm788 12368907

[19] Odeh M Sabo E Oliven A. Circulating levels of tumor necrosis factor-alpha correlate positively with severity of peripheral oedema in patients with right heart failure. Eur J Heart Fail. (2006) 8:141–6. 10.1016/j.ejheart.2005.05.010 16112904

[20] Kalantar-Zadeh K Anker SD Horwich TB Fonarow GC. Nutritional and anti-inflammatory interventions in chronic heart failure. Am J Cardiol. (2008) 101:89e−103. 10.1016/j.amjcard.2008.03.007 18514634 PMC5500213

[21] Suzuki S Hashizume N Kanzaki Y Maruyama T Kozuka A Yahikozawa K. Prognostic significance of serum albumin in patients with stable coronary artery disease treated by percutaneous coronary intervention. PLoS ONE. (2019) 14:e0219044. 10.1371/journal.pone.0219044 31269058 PMC6608965

[22] Fried LP Tangen CM Walston J Newman AB Hirsch C Gottdiener J. Frailty in older adults: evidence for a phenotype. J Gerontol A Biol Sci Med Sci. (2001) 56:M146–56. 10.1093/gerona/56.3.M146 11253156

[23] Clark AL Sze S. Impact of malnutrition using geriatric nutritional risk index in heart failure with preserved ejection fraction. JACC Heart Failure. (2019) 7:676–7. 10.1016/j.jchf.2019.06.002 31302047

[24] Cederholm T Barazzoni R Austin P Ballmer P Biolo G Bischoff SC. ESPEN guidelines on definitions and terminology of clinical nutrition. Clin Nutr. (2017) 36:49–64. 10.1016/j.clnu.2016.09.004 27642056

[25] Raposeiras Roubín S Abu Assi E Cespón Fernandez M Barreiro Pardal C Lizancos Castro A Parada JA. Prevalence and prognostic significance of malnutrition in patients with acute coronary syndrome. J Am Coll Cardiol. (2020) 76:828–40. 10.1016/j.jacc.2020.06.058 32792081

[26] Ragab MAA Galal SM Korany MA Ahmed AR. First derivative emission spectrofluorimetric method for the determination of LCZ696, a newly approved FDA supramolecular complex of valsartan and sacubitril in tablets. Luminescence. (2017) 32:1417–25. 10.1002/bio.3339 28569442

[27] Mullens W Auricchio A Martens P Witte K Cowie MR Delgado V. Optimized implementation of cardiac resynchronization therapy: a call for action for referral and optimization of care: a joint position statement from the Heart Failure Association (HFA), European Heart Rhythm Association (EHRA), and European Association of Cardiovascular Imaging (EACVI) of the European Society of Cardiology. Eur J Heart Failure. (2020) 22:2349–69. 10.1002/ejhf.2046 33136300

[28] Zelniker TA Braunwald E. Mechanisms of cardiorenal effects of sodium-glucose cotransporter 2 inhibitors: JACC state-of-the-art review. J Am Coll Cardiol. (2020) 75:422–34. 10.1016/j.jacc.2019.11.031 32000955

