# Corrigendum: Association of Malnutrition, Left Ventricular Ejection Fraction Category, and Mortality in Patients Undergoing Coronary Angiography: A Cohort With 45,826 Patients

**DOI:** 10.3389/fnut.2022.890490

**Published:** 2022-04-04

**Authors:** Ziling Mai, Zhidong Huang, Wenguang Lai, Huanqiang Li, Bo Wang, Sumei Huang, Yingming Shi, Sijia Yu, Qizheng Hu, Jin Liu, Lingyu Zhang, Yong Liu, Jiyan Chen, Yan Liang, Shilong Zhong, Shiqun Chen

**Affiliations:** ^1^School of Biology and Biological Engineering, South China University of Technology, Guangzhou, China; ^2^Department of Cardiology, Guangdong Provincial Key Laboratory of Coronary Heart Disease Prevention, Guangdong Cardiovascular Institute, Guangdong Provincial People's Hospital, Guangdong Academy of Medical Sciences, Guangzhou, China; ^3^Center of Scientific Research, Maoming People's Hospital, Maoming, China; ^4^Department of Cardiology, Maoming People's Hospital, Maoming, China; ^5^Department of Public Health, Guangdong Medical University, Dongguan, China; ^6^Department of Pharmacy, Guangdong Provincial People's Hospital, Guangdong Academy of Medical Sciences, Guangzhou, China

**Keywords:** malnutrition, left ventricular ejection fraction category, all-cause mortality, interaction, population attributable risk

In the published article, there was an error in affiliation 1. Instead of “Guangdong Provincial People's Hospital, School of Biology and Biological Engineering, South China University of Technology, Guangzhou, China”, it should be “School of Biology and Biological Engineering, South China University of Technology, Guangzhou, China”.

The authors apologize for this error and state that this does not change the scientific conclusions of the article in any way. The original article has been updated.

## Publisher's Note

All claims expressed in this article are solely those of the authors and do not necessarily represent those of their affiliated organizations, or those of the publisher, the editors and the reviewers. Any product that may be evaluated in this article, or claim that may be made by its manufacturer, is not guaranteed or endorsed by the publisher.

