# Supplementary material for: Association of Malnutrition, Left Ventricular Ejection Fraction Category, and Mortality in Patients Undergoing Coronary Angiography: A Cohort With 45,826 Patients
Source: Front Nutr. 2021 Sep 16;8:740746. doi: 10.3389/fnut.2021.740746 (PMC8481364; doi:10.3389/fnut.2021.740746)
Supplement: Supplementary Figure 1 — Flow of patients through the study. CONUT, Controlling Nutritional Status score; LVEF, Left ventricular ejection fraction. [file Presentation_1.PPTX]

## Slide 1
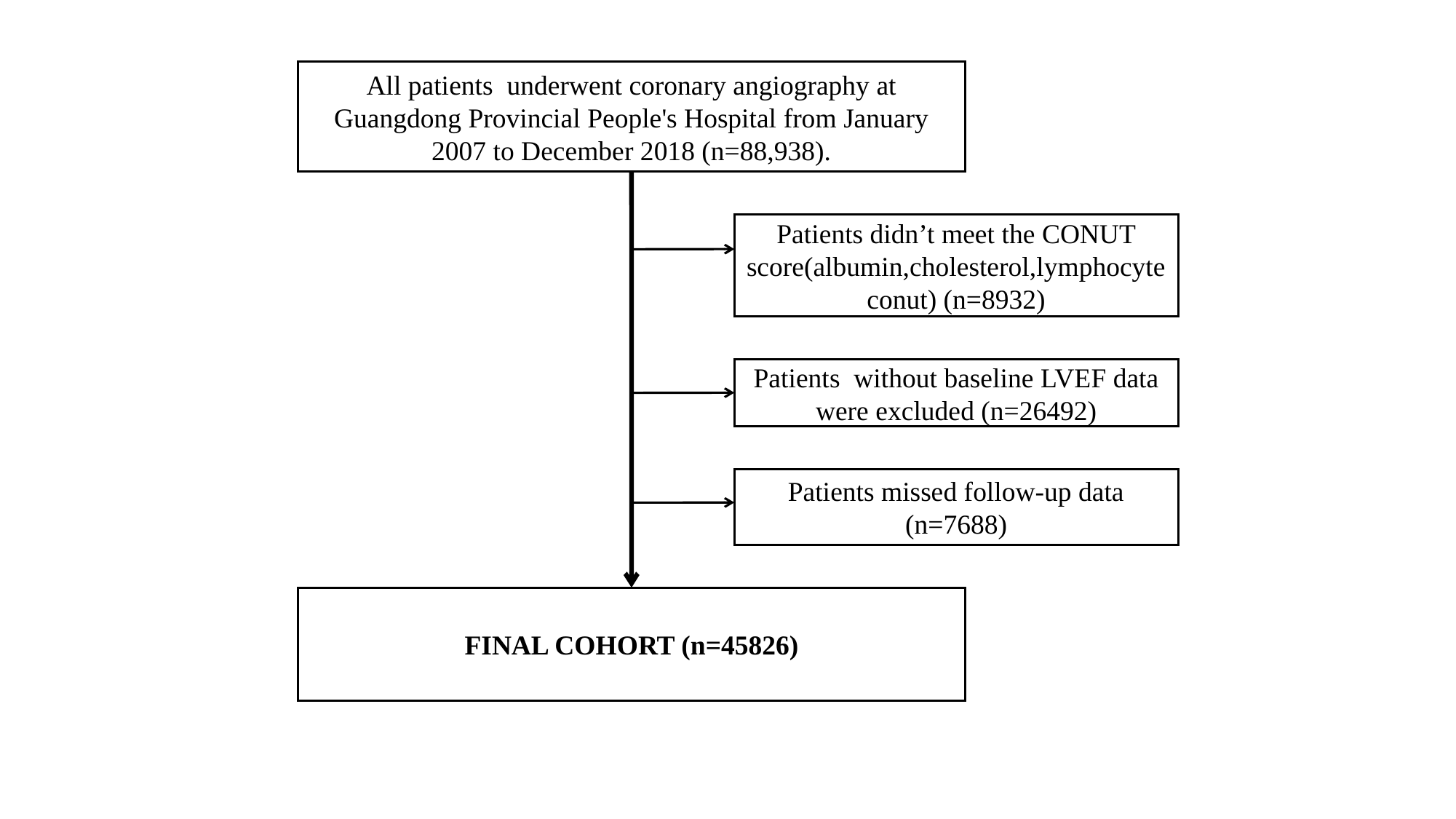

All patients underwent coronary angiography at Guangdong Provincial People's Hospital from January 2007 to December 2018 (n=88,938).
Patients didn’t meet the CONUT score(albumin,cholesterol,lymphocyte conut) (n=8932)
Patients without baseline LVEF data were excluded (n=26492)
Patients missed follow-up data (n=7688)
FINAL COHORT (n=45826)
