# Supplementary material for: Association of Malnutrition, Left Ventricular Ejection Fraction Category, and Mortality in Patients Undergoing Coronary Angiography: A Cohort With 45,826 Patients
Source: Front Nutr. 2021 Sep 16;8:740746. doi: 10.3389/fnut.2021.740746 (PMC8481364; doi:10.3389/fnut.2021.740746)
Supplement: Supplementary Table 1 — Hazard ratios for all-cause mortality stratified by the nutritional state in patients with LVEF ≥ 40% and LVEF < 40%. [file Table_1.docx]

|  | | | LVEF<40% | | |  | LVEF≥40% | |
| --- | --- | --- | --- | --- | --- | --- | --- | --- |
| Factors | HR (95% CI) | | | | P-value |  | HR (95% CI) | P-value |
| Normal | | Ref | |  | |  | Ref |  |
| Mild | | 1.00(0.83-1.21) | | 0.98 | |  | 1.21(1.12-1.31) | <0.001 |
| Moderate | | 1.20(0.95-1.51) | | 0.124 | |  | 1.56(1.40-1.74) | <0.001 |
| Severe | | 1.41(0.87-2.29) | | 0.164 | |  | 2.20(1.67-2.9) | <0.001 |
| Age, year | | 1.01(1.00-1.02) | | 0.001 | |  | 1.02(1.02-1.02) | <0.001 |
| Female | | 0.91(0.75-1.10) | | 0.314 | |  | 0.84(0.79-0.90) | <0.001 |
| Hypertension | | 0.94(0.81-1.10) | | 0.433 | |  | 1.00(0.94-1.07) | 0.967 |
| Diabetes mellitus | | 1.18(1.01-1.38) | | 0.043 | |  | 1.21(1.12-1.30) | <0.001 |
| Percutaneous coronary intervention | | 0.78(0.65-0.93) | | 0.005 | |  | 0.88(0.81-0.96) | 0.003 |
| Coronary artery disease | | 1.19(0.92-1.53) | | 0.185 | |  | 1.15(1.04-1.27) | 0.005 |
| Chronic kidney diseases | | 1.43(1.22-1.67) | | <0.001 | |  | 1.49(1.38-1.61) | <0.001 |
| Atrial fibrillation | | 1.01(0.77-1.32) | | 0.939 | |  | 1.17(1.05-1.30) | 0.005 |
| Anemia | | 1.20(1.02-1.41) | | 0.026 | |  | 1.26(1.18-1.35) | <0.001 |
| Stroke | | 1.00(0.74-1.36) | | 0.979 | |  | 1.21(1.07-1.37) | 0.002 |
| Low-density lipoprotein cholesterol, mmol/L | | 1.00(0.92-1.09) | | 0.946 | |  | 1.04(1.00-1.08) | 0.035 |
| High-density lipoprotein cholesterol, mmol/L | | 0.74(0.55-1.00) | | 0.047 | |  | 0.99(0.88-1.11) | 0.856 |
| Triglycerides | | 1.03(0.94-1.12) | | 0.586 | |  | 0.97(0.94-1.00) | 0.096 |
| Statins | | 0.91(0.73-1.15) | | 0.434 | |  | 0.77(0.70-0.84) | <0.001 |

**Supplemental Table 1. Hazard ratios for all-cause mortality stratified by the nutritional state in patients with LVEF ≥ 40% and LVEF<40%.**
